# New insights into *Gongylonema* infections in exotic mammals: description of *G. (G.) primatum* sp. nov. and detection of zoonotic *G. pulchrum*

**DOI:** 10.3389/fvets.2026.1862920

**Published:** 2026-07-08

**Authors:** Ondřej Máca, David González-Solís

**Affiliations:** 1 Department of Zoology and Fisheries, Faculty of Agrobiology, Food and Natural Resources, Czech University of Life Sciences Prague, Prague, Czechia; 2 Department of Pathology and Parasitology, State Veterinary Institute Prague, Prague, Czechia; 3 Department of Systematics and Aquatic Ecology, El Colegio de la Frontera Sur, Chetumal, Quintana Roo, Mexico

**Keywords:** Czech Republic, molecular and phylogenetic analysis, morphology, new species, primates, zoonotic

## Abstract

This study aimed to identify nematodes of the genus *Gongylonema* using morphological and molecular methods and to determine their occurrence in a group of non-human primates and anteaters. A single female nematode collected from *Macaca sylvanus*, together with carcasses of 29 non-human primates (belonging to 13 species) and two anteaters (representing one species) from Czech zoological gardens and private collections, were submitted between 2019 and 2025 to the State Veterinary Institute Prague for necropsies. Smears from the buccal cavity and tongue, as well as fecal samples, were collected by veterinarians at each facility and transported to the laboratory in tubes containing saline solution or in plastic bags, respectively. The samples were genetically characterized using the nuclear *18S* rRNA, *28S* rRNA genes, the internal transcribed spacer (ITS) region, and the mitochondrial cytochrome c oxidase subunit 1 gene (*cox1*). Four non-human primates (two *Pithecia pithecia*, one *Saguinus labiatus*, and one *S. midas*) and two anteaters (*Tamandua tetradactyla*), from the same zoological garden, were positive for *Gongylonema (G.) primatum* sp. nov. The single female specimen from *M. sylvanus* was identified as *G. pulchrum*. The new species morphologically differed from its congeners in the body length of adult males, the left spicule length, the gubernaculum length, the protruded vulvar lips, and the number of precloacal papillae. Molecularly, the *18S* and *28S* rRNA genes were 99.88−100% similar to those of *G. nepalensis* and *G. pulchrum*, whereas the ITS and *cox1* sequences showed lower similarity to those of both *Gongylonema* species. *Gongylonema primatum* sp. nov. was morphologically and molecularly different from its congeners and therefore represents a new species. This is the first record of the zoonotic *G. pulchrum* in *M. sylvanus*. Although *G. pulchrum* is zoonotic, the new species may also have zoonotic potential.

## Introduction

*Gongylonema* Molin, 1857 is one of the spirurid genera of high veterinary and medical importance. This genus comprises 47 species of parasitic nematodes inhabiting the mucosal epithelium of the mouth, esophagus, and stomach of mammals (including humans) and birds (1–3), where they can cause various disorders.

Within mammals, members of the orders Rodentia and Primates are the most common hosts for these nematodes, whereas members of the order Pilosa (which includes anteaters) have not previously been reported as hosts of *Gongylonema* species. In Primates, gongylonemosis caused by *G*. *capucini* Maplestone, 1939; *G*. *macrogubernaculum* Lubimov, 1931; *G*. *microgubernaculum* Gebauer, 1933; *G. pulchrum* Molin, 1857; and *G*. *saimirisi* Artigas, 1933 has been reported mainly in the palate, lips, and tongue of various non-human primates worldwide (including the genera *Callimico*, *Callithrix*, *Cebus*, *Cercopithecus*, *Leontopithecus*, *Macaca*, *Papio*, *Saguinus*, and *Saimiri*). However, in some cases, the nematodes were not identified to the species level or were assigned to *G. pulchrum* [e.g., (4–11)], which is responsible for all reported cases of gongylonemosis in humans worldwide [see (12)]. Among the above-mentioned reports, at least one case involved the possible transmission of nematodes between two co-housed host species, squirrels and non-human primates, kept in the same area or in close proximity [see (6)].

Even though there are reports of *Gongylonema* species in the above-mentioned genera of non-human primates, there is always a possibility of finding additional nematode species in the same host species from different locations. Additionally, those *Gongylonema* found in *Leontopithecus* were only reported to the genus level, whereas these nematodes have never been reported in members of *Cebuella* and *Pithecia*. In our study, non-human primates of various species and anteaters were thoroughly examined to determine the presence and identity of nematodes using morphological and molecular methods. In addition, a single female nematode was examined to determine its genetic identity. Thus, this study aimed to identify the nematodes using morphological and molecular methods and to determine their presence in a group of non-human primates and anteaters, some of which cohabited in the same captive facilities.

## Methods

An individual female nematode was found in the esophagus of a dead Barbary macaque, *Macaca sylvanus* (Linnaeus, 1758), which was sent to the State Veterinary Institute Prague (SVIP) for molecular identification (this specimen was not morphologically examined). Additionally, 29 non-human primates that died due to unknown causes or poor nutritional status were sent between 2019 and 2025 to the SVIP for necropsies and parasitological examinations of organs and tissues, such as the common marmoset *Callithrix jacchus* (Linnaeus, 1758), the white-headed marmoset *Callithrix geoffroyi* (Humboldt, 1812), the eastern pygmy marmoset *Cebuella niveiventris* Lönnberg, 1940, the Colombian white-faced capuchin *Cebus capucinus* (Linnaeus, 1758), the golden lion tamarin *Leontopithecus rosalia* (Linnaeus, 1766), the Celebes crested macaque *Macaca nigra nigra* (Desmarest, 1822) (*n* = 1 each); the western pygmy marmoset *Callithrix pygmaea* (Spix, 1823), the golden-headed lion tamarin *Leontopithecus chrysomelas* (Kuhl, 1820), the white-faced saki *Pithecia pithecia* (Linnaeus, 1766), the Guianan squirrel monkey *Saimiri sciureus* (Linnaeus, 1758) (*n* = 2 each); the white-lipped tamarin *Saguinus labiatus* (Geoffroy in Humbolt, 1812), the cotton-top tamarin *Saguinus oedipus* (Linnaeus, 1758) (*n* = 3 each); the Midas tamarin *Saguinus midas* (Linnaeus, 1758) (*n* = 9); as well as two collared anteaters *Tamandua tetradactyla* (Linnaeus, 1758) from private breeders and Czech zoological gardens. All examined non-human primates and anteaters were opportunistically obtained and were not collected as part of a systematic study of these hosts.

**Figure 1:**
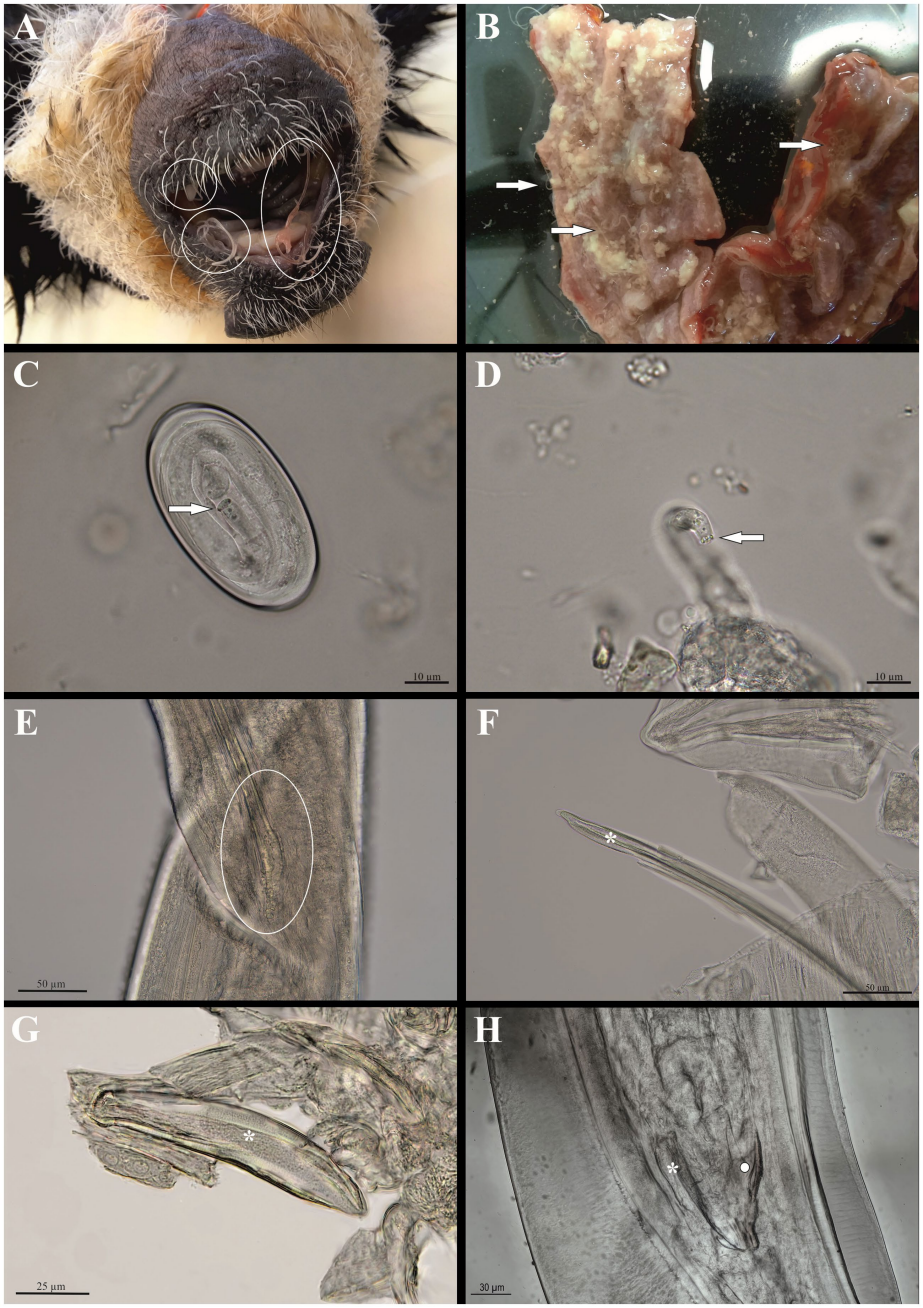
White-faced saki *Pithecia pithecia* (Linnaeus, 1766), **(A,B)** Head with some nematodes in the buccal cavity (circles) and esophagus (arrows), respectively. *Gongylonema* (*G*.) *primatum* sp. nov. light microscopy: **(C)** Mature egg (arrow indicates tail tip of larva), **(D)** Tail of released larva with mucrons on tip (arrow), **(E)** proximal end of left spicule (circle), **(F)** Distal end of left spicule (asterisk), **(G)** Right spicule (asterisk), **(H)** Right spicule (asterisk) and gubernaculum (white dot).

Some smears from the buccal cavity and tongue, as well as fecal samples, were collected by veterinarians at each facility and transported to the laboratory in tubes containing saline solution or in plastic bags, respectively. Once at the SVIP and after macroscopical necropsy, 26 samples, including smears of the buccal cavity, tongue, and esophagus; feces (Figure 1B); and intestinal samples, were examined by wet mounts or flotation–centrifugation coprological methods. Nematodes were stored in 70% ethanol and 10% formalin for molecular and morphological identification, respectively, and were cleared with a mixture of glycerin and water for physical examination. For scanning electron microscopy, they were dehydrated through an ethanol series, critical-point-dried, and sputter-coated with gold. Finally, they were examined using a scanning electron microscope (SEM) (JEOL Model JSM6010, JEOL, Akishima, Tokyo, Japan) at El Colegio de la Frontera Sur (ECOSUR) Chetumal Unit. Type specimens were preserved in a mixture of ethanol–glycerin or on SEM stubs and deposited in the reference collection of ECOSUR in Mexico (ECOPA−137H, 137A, 137P). Drawings were made with the aid of an Olympus drawing tube attached to an Olympus CX31 microscope. Larvae were manually released from eggs to study their morphology (Figure 1D). Nematodes and eggs were observed and photographed by light microscopy using Leica DMLB and Leica DM2500 LED optical microscopes equipped with Leica DFC420 and Leica DMC5400 digital cameras, and Leica Application Suite X microscope software (Leica Microsystems, Wetzlar, Germany). Measurements are given in micrometers, unless otherwise indicated. The taxonomic classification of nematodes follows that of Hodda (13), and their specific identity was based on Baylis (14) and Chabaud (15).

Nematodes collected from the buccal cavity (Figure 1A), tongue, or esophagus were placed separately in 1.5-mL Eppendorf tubes containing 70% ethanol and were molecularly analyzed within 1−90 days after collection. The genomic DNA from every single nematode (whole nematode, anterior end of male or female) was extracted using the NucleoSpin tissue XS kit (Macherey-Nagel, Düren, Germany) according to the manufacturer’s protocol and stored at −20 °C. Conventional polymerase chain reaction (PCR) assays were used for confirmation of the identity of nematodes focused on nuclear *18S* rRNA (18S fwd + 18S rev or op18SF/op18SR + Op18SR/OP18SIntF) (16, 17) and *28S* rRNA genes (28S fwd + 28S rev) (18), internal transcribed spacer (ITS) region (partial ITS1, complete *5.8S* and ITS2, partial *28S*; 5fwd + 5rev) (16), and mitochondrial cytochrome c oxidase subunit 1 (*cox1*; JB3 + JB4.5 or/and coxntf+ntr/coxntf+int) (19, 20) markers (Table 1). PCR or nested PCR was performed in total volumes of 25 μL containing 12.50 μL of GoTaq^®^ G2 Green Master Mix (Promega, Madison, Wisconsin, USA), 0.4 μM of each primer, 5 μL of DNA template, and PCR-grade water. The thermal cycler conditions were set at an initial denaturation at 95 °C for 3 min, followed by 30 cycles of amplification (95 °C for 30 s, 55–60 °C for 30 s, and 72 °C for 1 min), as well as a final extension step at 72 °C for 10 min; negative (nuclease-free water) controls were included in each PCR assay. The PCR products were electrophoresed in a 1.0% (w/v) agarose gel with ethidium bromide stain. Subsequently, PCR products were purified with ExoSAP-IT™ Express PCR Product Cleanup Reagent kit (Thermo Fisher Scientific, Waltham, MA, USA) and sequenced in both directions by the commercial company Eurofins Genomics (Ebersberg, Germany). New sequences and those of closely related gongylonematids found in the National Centre for Biotechnology Information (NCBI) database^1^ were compared with the Basic Local Alignment Search Tool^2^ on the NCBI website and submitted to GenBank. Molecular Evolutionary Genetics Analysis software version 12.0.11 (21) was used to compare the amplified sequences. Phylogenetic trees were inferred based on the maximum likelihood method and the best-fit models. The Tamura 3-parameter model with gamma-distributed rate variation and invariant sites (T92 + G + I) was selected for the *18S* rRNA dataset, which comprised 40 nucleotide sequences with 1,766 positions in the final alignment and was rooted using *Toxocara canis* (JN256976). The General Time Reversible model with gamma-distributed rate variation and invariant sites (GTR + G + I) was used for the *cox1* gene, which comprised 31 nucleotide sequences with 903 positions and was rooted using *Mansonella ozzardi* (NC082197) and *Mansonella perstans* (OQ633019). It was also used for the ITS, which encompassed 19 nucleotide sequences with 2,284 positions, rooted using *Acanthocheilonema reconditum* (HG964684). The reliability of the trees was estimated by 1,000 bootstrap replications.

**Table 1:** List of primers used for targeting four loci used for identifying *Gongylonema* species in several host species.

Target locus	Primer name	Sequence (5–3′)	Temp. (°C)	Size (bp)	Reference
*18S* rRNA	18S fwd	TCCAAGGAAGGCAGCAGGC	60	~1,140	16
	18S rev	CGACGGGCGGTGTGTACA			
	Op18SF	CCGATTGATTCTGTCGGCGGTTA		~1,200	17
	OP18SR	CACCTACGGAAACCTTGTTACGAC			
	OP18SIntF	CTCAACACGGGAAAACTCACCTG			
*28S* rRNA	28FWD	GGGAAAGAAGACCCTGTTGAG		~720	18
	28REV	TTCTGACTTAGAGGCGTTCAG			
ITS (*18S*-ITS1-*5.8S*-ITS2-*28S*)	5FWD	AGGTGAACCTGCGGAAGGATCATT	58	~1,100	16
	5REV	TTCACGCCCTCTTGAACTCT			
*cox1*	Cox JB3	TTTTTTGGGCATCCTGAGGTTTAT	55	~410	19
	Cox JB4.5	TAAAGAAAGAACATAATGAAAATG			
	Cox1NTF	TGATTGGTGGTTTTGGTAA	56	~650	20
	Cox1NTR	ATAAGTACGAGTATCAATATC			
	Cox1NTInt	GGCTAGACAACTCTAAACG			

## Results

Out of the 29 non-human primates and two anteaters examined, four non-human primates (two *P. pithecia*, one *S. labiatus*, one *S. midas*) and the two anteaters (*T. tetradactyla*) from the same zoological garden were positive for *Gongylonema (G.) primatum* sp. nov. in the buccal cavity, tongue, and esophagus (Figure 1B). It was not possible to count all nematodes, but *S. labiatus* and *S. midas* harbored very few individuals (3 to 5), *T. tetradactyla* had more (10 to 20), whereas *P. pithecia* was much more parasitized (10 to 50 specimens). Moreover, the single female nematode in the esophagus of *M. sylvanus* from another zoological garden belonged to the species *G. pulchrum*. On the other hand, *C. geoffroyi*, *C. jacchus*, *C. pygmaea*, *Ce*. *niveiventris*, *Ceb*. *capucinus*, *L. chrysomelas*, *L. rosalia*, *M*. *n*. *nigra*, *S. oedipus*, and *Sai*. *sciureus* were free of gongylonematids.

Three smears from the buccal cavity, esophagus, and tongue of *P. pithecia*, *S. labiatus*, and *T. tetradactyla* were positive for the presence of *Gongylonema* spp. eggs. In all cases, the eggs were oval, larvated, and had a thick, smooth shell (Figure 1C). First-stage larvae were elongated, with a crown of murons on the tail tip (Figure 1D). Postmortem coprological analysis showed that only *P. pithecia* and *T. tetradactyla* were positive for *Gongylonema* eggs in the intestinal content and rectum, whereas *M. sylvanus*, *S. labiatus*, and *S. midas* were negative.

### Taxonomic summary

Family Gongylonematidae Hall, 1916.

Genus *Gongylonema* Molin, 1857.

Subgenus *Gongylonema* Molin, 1857.

**Figure 2:**
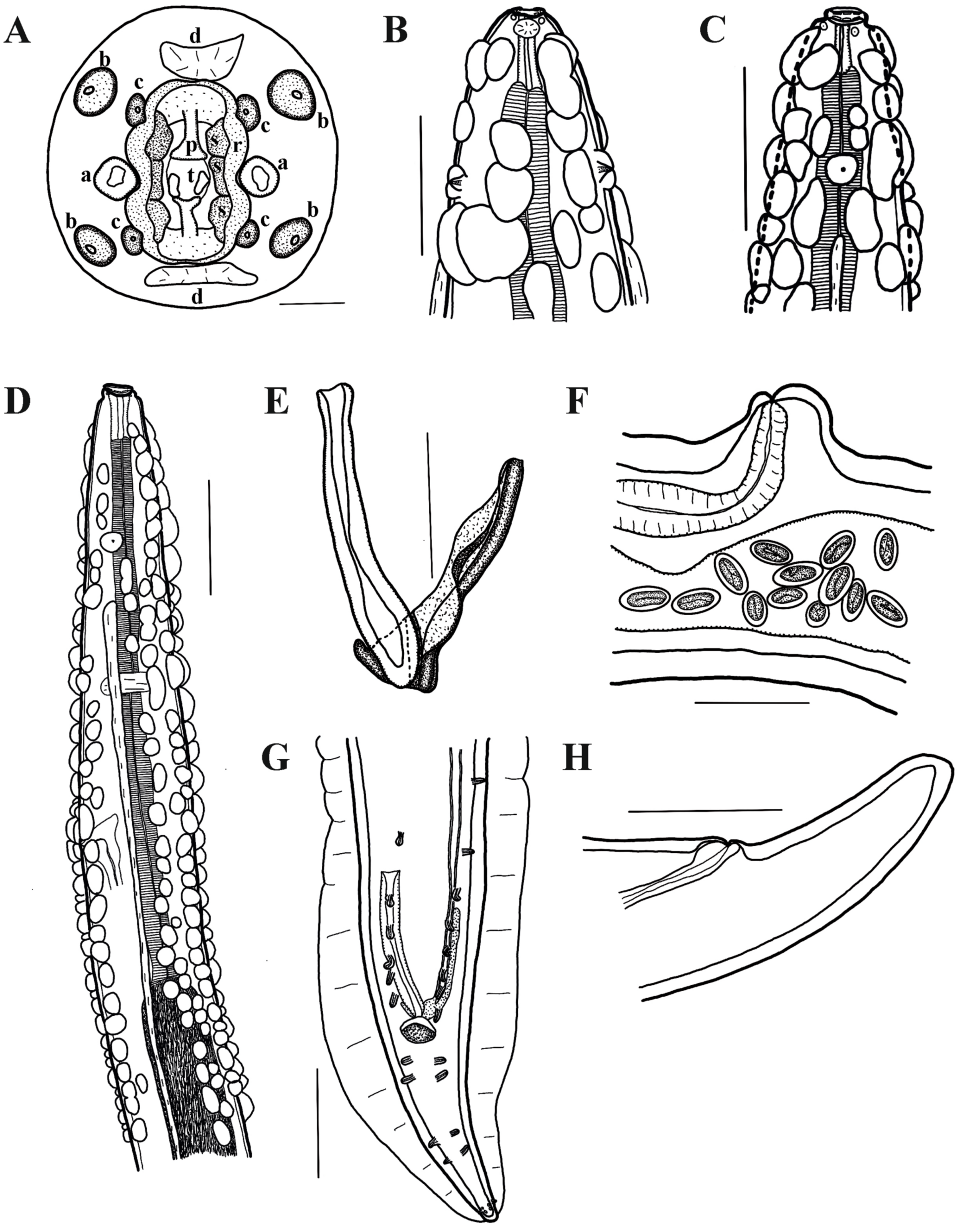
*Gongylonema* (*G*.) *primatum* sp. nov. line drawings. **(A)** Cephalic end of male, apical view. **(B,C)** Cephalic end of male, dorsoventral and lateral views, respectively. **(D)** Anterior end of female body, lateral view. **(E)** Right spicule and gubernaculum, ventral view. **(F)** Region of the vulva, lateral view. **(G)** Posterior end of male, ventral view. **(H)** Posterior end of female, lateral view. Scale bars: a = 5 μm, b–d, f–h = 100 μm, e = 50 μm. a, amphid; b, outer papilla; c, inner papilla; d, depression; p, pseudolabium; r, rim; s, sublabium; t, tooth.

**Figure 3:**
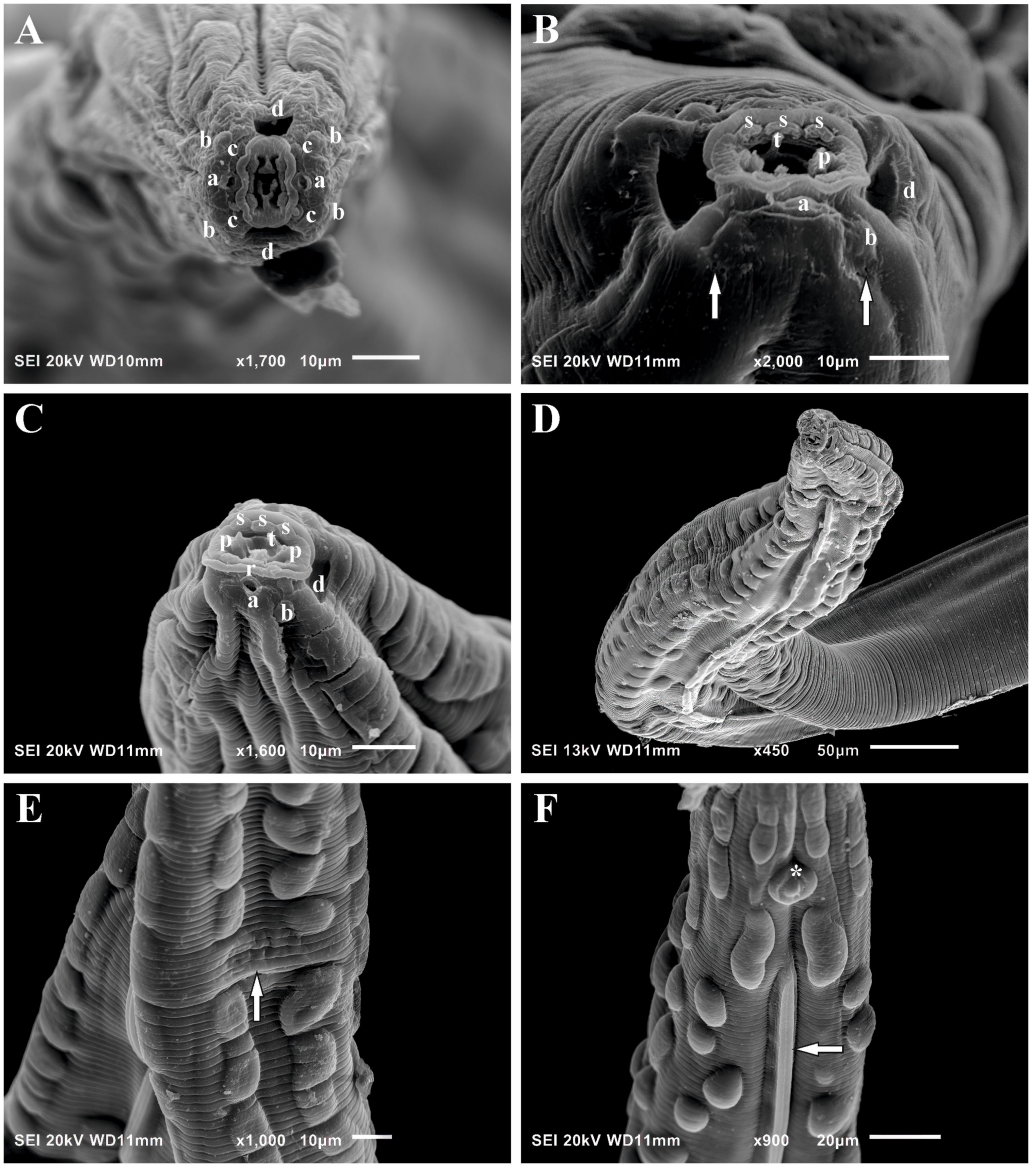
*Gongylonema* (*G*.) *primatum* sp. nov. scanning electron micrographs. **(A)** Cephalic end of female, apical view. **(B)** Cephalic end of male, subapical view (arrows indicate pores below outer papilla). **(C)** Cephalic end of male, subapical view (another specimen). **(D)** Anterior end of the male body. **(E)** Region of excretory pore, subventral view (arrow indicates excretory pore outlet). **(F)** Region of cephalic papilla (asterisk) and lateral alae (arrow), lateral view. a, amphid; b, outer papilla; c, inner papilla; d, depression; p, pseudolabium; r, rim; s, sublabium; t, tooth.

**Figure 4:**
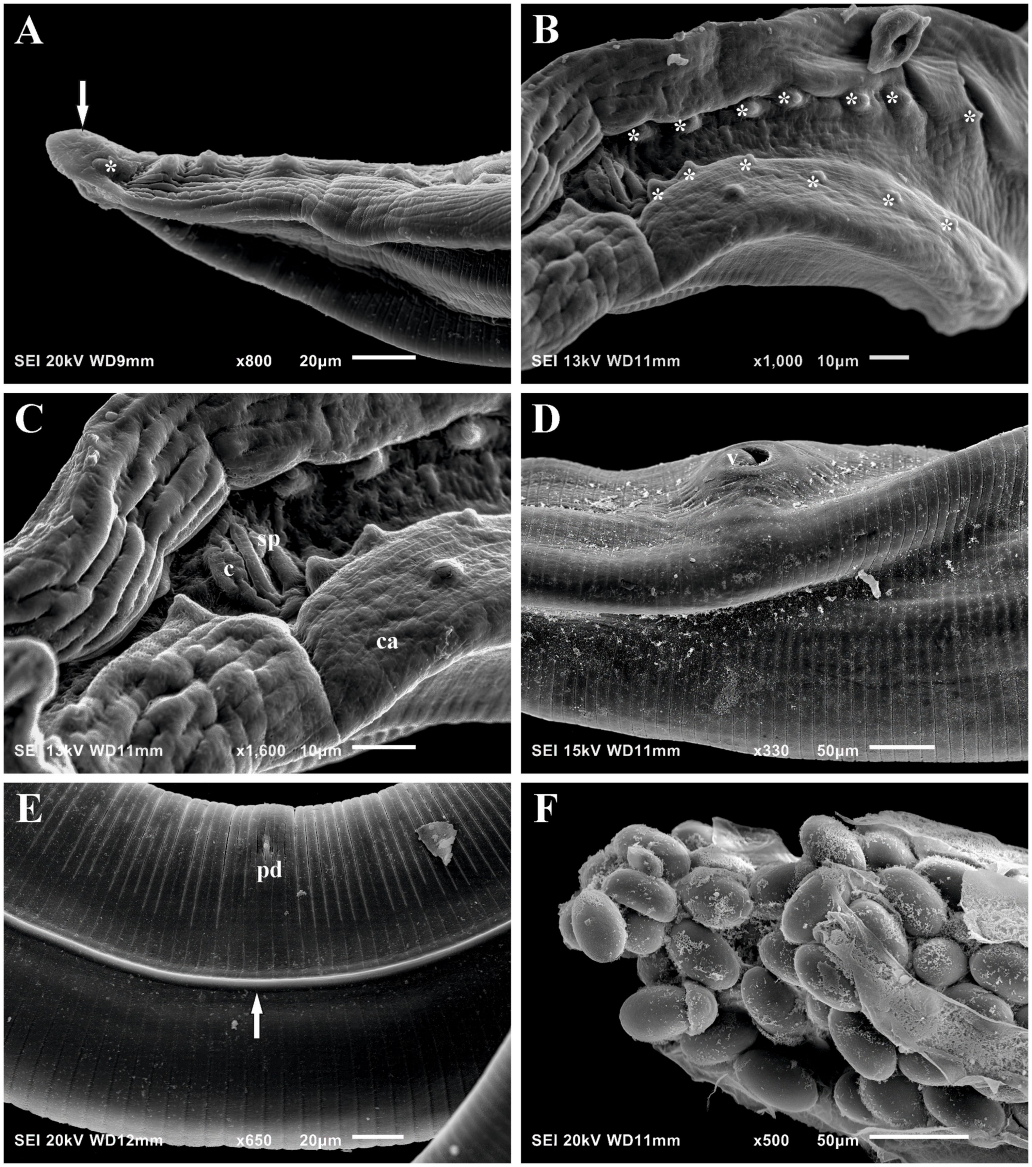
*Gongylonema* (*G*.) *primatum* sp. nov. scanning electron micrographs. **(A)** Caudal end of male, lateral view (asterisk indicates lateral papilla, arrow indicates pair of flattened ventral papillae). **(B)** Caudal end of male, ventral view (asterisks indicate precloacal papillae). **(C)** Region of cloaca, ventral view. **(D)** Region of the vulva, lateral view. **(E)** Postdeirid, lateral view (arrow indicates lateral alae). **(F)** Eggs in the uterus. c, cloaca; ca, caudal alae; pd., postdeirid; sp., spicule; v, vulva.

**Figure 5:**
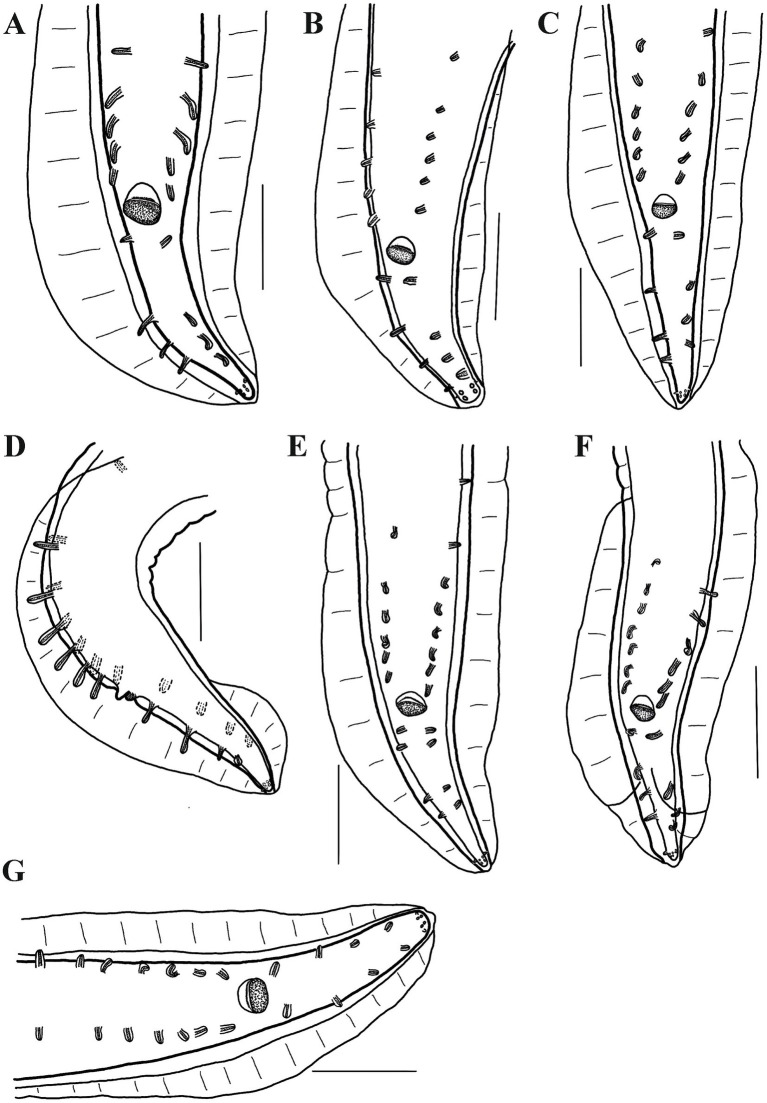
*Gongylonema* (*G*.) *primatum* sp. nov. line drawings. Posterior end of male with different combinations (left/right side) of caudal papillae. **(A)** 5/5 from *Pithecia pithecia*, ventral view. **(B)** 5/6 from *Saguinus midas*, subventral view. **(C)** 6/6 from *Tamandua tetradactyla*, ventral view. **(D)** 6/7 from *Tamandua tetradactyla*, sublateral view. **(E)** 6/8 from *Tamandua tetradactyla*, ventral view. **(F)** 7/6 from *Pithecia pithecia*, ventral view. **(G)** 7/7 from *Tamandua tetradactyla*, ventral view. Scale bars: a–g = 100 μm.

*Gongylonema* (*Gongylonema*) *primatum* sp. nov. (Figures 1–5 and Table 2).

**Table 2:** Morphometrics of the mature nematodes of *Gongylonema (Gongylonema) primatum* sp. nov. in different hosts from the Czech Republic.

Morphological character	*Pithecia pithecia*		*Saguinus labiatus*	*Saguinus midas*		*Tamandua tetradactyla*	
	3♂	5♀	♂	♂	♀	9♂	6♀
Body length (mm)	15.35–21.55	19.60–57.40	16.02	11.10	18.80	11.70–17.15	22.42–29.72
Maximum width	150–200	175–300	132	132	204	132–173	183–225
Distance of nerve ring	204–275	255–336	226	206	236	196–265	295–326
Distance of deirids	79–134	71–174	114	99	109	92–139	131–154
Distance of excretory pore	336–459	428–652	398	346	408	336–459	499–520
Vestibule length	34–47	32–59	39	37	37	35–47	44–71
Muscular esophagus length	428–499	465–642	489	387	520	387–499	571–612
Glandular esophagus length (mm)	3.36–4.99	4.98–7.12	3.89	3.27	4.20	3.40–4.28	4.71–5.42
Rigth spicule length	82–104		112	92		84–110	
Left spicule length (mm)	2.31–4.92		3.46	2.85		3.14–3.80	
Gubernaculum length	67–77		72	67		62–87	
Tail length	153–255	153–224	191	151	144	164–20	142–204
Precloacal combinations (right/left side)	5/5, 5/6, 6/6,7/6 (young adult)		7/7	7/6		6/6, 6/7, 6/8, 7/7, 7/6	
Adcloacal	0		0	0		0	
Postcloacal	7		7	7		7	
Distance of vulva (mm)		18.25–54.40			17.30		20.67–27.62
Egg length Egg width		42–54 17–31			42–44 24–27		39–52 19–32
Distance of postdeirid (mm)	10.27	6.42–22.20	6.47	4.42	6.98	5.75–8.55	10.22–11.87

Numbers next to each gender symbol represent the examined specimens. Measurements in micrometers, unless otherwise stated.

All distances were taken from the anterior body end.

Description: Medium-sized nematodes, whitish in color. Anterior end rounded, posterior end conical with a rounded tip. Cuticle transversely striated, with prominent cuticular, oval or circular bosses irregularly arranged in longitudinal rows, starting at the level of the vestibule and ending slightly posterior to the junction of the muscular and glandular esophagus (Figures 2D, 3D). Lateral alae starting at the level of the nerve ring (Figures 2C,D, 3F) and extending up to the last third of the body. Mouth opening oval, surrounded by a sclerotized circumoral rim, with four single submedian papillae on the inner circle, four single submedian papillae on the outer circle, and a pair of lateral amphids (Figures 2A, 3A–C). Mouth armed with six lateral labia, two bifurcated dorsoventral interlabia, and two lateral tooth-like structures. Two large (dorsal and ventral) depressions external to the sclerotized circumoral rim (Figures 2A, 3A–C). Vestibule funnel-shaped and well sclerotized (Figures 2B,C). Esophagus divided into muscular and glandular parts, the latter much longer and broader than the former. Excretory pore slit-like, located between the nerve ring and the junction of the muscular and glandular esophagus (Figures 2D, 3E). Deirids lateral, relatively large, spike-like with a globose base (similar to a cuticular boss), situated slightly anterior to the beginning of lateral alae (Figures 2B,C, 3F). Postdeirid subventral, spike-like (Figure 4E), located slightly pre-equatorial. Tail conical, with rounded tip (Figure 4A).

Male (holotype from *Tamandua tetradactyla*, paratypes from different hosts in parentheses, see Table 2): Body length 17.15 (11.10–21.55) mm; maximum width 132 (132–200). Distance of nerve ring, excretory pore, and deirids 259 (196–275), 428 (336–459), and 129 (79–139), respectively, from the anterior end of the body. Vestibule 37 (34–47) long. Length of muscular esophagus 469 (387–499) and glandular part 3.72 (3.27–4.99) mm. Distance of unpaired papilla (postdeirid) 6.87 (4.42–10.27) mm from the anterior body end. Testes reaching the anteriorly last third of the glandular esophagus. Caudal alae present, extended up to the tail tip (Figure 2G). Caudal papillae: 6 (5–7) subventral pedunculated precloacal pairs on right side, 7 (5–8) on left side; 7 (7) postcloacal pairs, first four pairs of postcloacals subventral and pedunculated, one lateral, and last two pairs ventral, small and flattened (Figures 2G, 4A, 5A–G). Phasmids small, pit-like, lateral, near the tail tip. Spicules unequal, dissimilar; left spicule 3.22 (2.31–4.92) mm long, slender, with short handle and very long lamina, proximal end with small granular outgrows (Figure 1E), distal end with small, orbicular, transparent membranous structure (Figure 1F); right spicule thick, boat-shaped, short, 102 (82–112) long, with broad proximal end and rounded distal end (Figure 1G). Gubernaculum slightly smaller than right spicule, spoon-like, 62 (62–87) long, with left edge and distal end more sclerotized than the rest of the gubernaculum (Figures 1H, 2E). Cloaca with slightly elevated lips (Figures 4C, 5D). Tail ventrally bent, 204 (151–255) long.

Fully gravid female (allotype from *T. tetradactyla*, paratypes from several hosts in parentheses, see Table 2): Body length 29.30 (18.80–57.40) mm; maximum width 183 (175–300). Distance of nerve ring, excretory pore, and deirids from the anterior end of the body 326 (236–336), 510 (408–652), and 154 (71–174), respectively. Vestibule 71 (32–71) long. Length of muscular esophagus 581 (465–642); glandular part 4.96 (4.20–7.12) mm in length. Distance of unpaired papilla (postdeirid) from the anterior body end 11.87 (6.42–22.20) mm. Vulva slit-like, slightly anterior to anus, 27.57 (17.30–54.40) mm, from anterior body end, with protruded lips (Figures 2F, 4D). Vagina directed anteriorly. Amphidelphic, uterus reaching anteriorly, posterior to the junction of the esophagus and intestine, and posteriorly to the level of the anus. Larvated eggs are thick-walled (Figures 1C, 4F), 44–49 (39–54) long and 27–29 (17–32) wide. Tail (Figure 2H) 153 (142–224) long.

Gravid female (based on one specimen from *T. tetradactyla*): Body length 18.00 mm; maximum width 142. The distance of the nerve ring is 263, and the excretory pore is 400, both from the anterior end of the body. Deirids not visible. Vestibule 42 long. Length of muscular esophagus 498; glandular part 4.48 mm long. Postdeirid not visible. Vulva slit-like, slightly anterior to the anus, 16.67 mm from anterior body end, with protruded lips. Vagina directed anteriorly. Poorly developed eggs are oval with a thin and smooth shell, 14–17 long and 10–12 wide. Tail 129 long.

Non-gravid female (based on one specimen from *T. tetradactyla*): Body length 13.07 mm; maximum width 142. Distance of nerve ring 219, excretory pore 357, and deirids 99, from the anterior end of the body. Vestibule 34 long. Length of muscular esophagus 408; glandular part 3.30 mm long. Postdeirid not visible. Vulva slit-like, slightly anterior to anus, 12.15 mm from anterior body end, with protruded lips. Vagina directed anteriorly. Tail length 131.

Late first-stage or early second-stage larvae from *Pithecia pithecia* (based on three specimens): Body length 1.55–1.98 mm; maximum width 51–61. Distance of nerve ring, excretory pore, and deirids 89–129, 129–204, and 42, respectively, from the anterior end of the body. Vestibule 24–29 long. Length of muscular esophagus 146–211, glandular part 836–1,264. Distance of unpaired papilla (postdeirid) 989–1,417, from anterior body end. Tail 67–74 long, with a crown of small mucrons on the tail tip (Figure 1D).

Third-stage larvae from *Pithecia pithecia* (based on seven specimens): Body length 2.97–5.40 mm; maximum width 71–91. Distance of nerve ring, excretory pore, and deirids 142–171, 214–295, and 62–94, respectively, from the anterior end of the body. Vestibule 24–37 long. Length of muscular esophagus 229–326, glandular part 1,683–2,356. Distance of unpaired papilla (postdeirid) 2.20–4.26 mm from the anterior body end. Tail 91–112 long, without mucrons on the tail tip.

Type host: Collared anteater *Tamandua tetradactyla* (Linnaeus, 1758) (Mammalia, Pilosa).

Other hosts: White-faced saki *Pithecia pithecia* (Linnaeus, 1766), white-lipped tamarin *Saguinus labiatus* (Geoffroy in Humbolt, 1812), Midas tamarin *Saguinus midas* (Linnaeus, 1758) (all Mammalia, Primates).

Site of infection: Buccal cavity, tongue, and esophagus.

Type locality: Zoological Garden in the Czech Republic.

Deposition of material: Holotype accession number (ECOPA−137H), allotype accession number (ECOPA−137A), paratype accession numbers (ECOPA−137P) (males and females) were deposited at the reference collection of ECOSUR. All sequence data generated were deposited in the GenBank database: *18S* rRNA (PZ134051), *28S* rRNA (PZ134052), *cox1* (PZ126580), and ITS (PZ134053).

ZooBank registration: To comply with the regulations set out in Article 8.5 of the amended 2012 version of the International Code of Zoological Nomenclature (22), details of the new species have been submitted to ZooBank. The Life Science Identifier (LSID) is urn: lsid:zoobank.org:pub: BC8A8316-ED1C-4A31-9611-083E94A81F52.

Etymology: The species epithet is related to the group of mammals used as hosts (i.e., primates).

Remarks: Out of the five known *Gongylonema* species reported in non-human primates, the new species differs from *G*. *capucini* in the body length of adult males (11.10–21.55 vs. 5.40–6.30 mm) and left spicule (2.31–4.92 vs. 0.520 mm), from *G*. *macrogubernaculum* and *G*. *microgubernaculum* in the left spicule length (5.00–6.00 and 2.08 mm, respectively) and gubernaculum length (62–87 vs. 165 and 45 μm, respectively), from *G. pulchrum* in the left spicule (7.54 mm), whereas *G*. *saimirisi* has similar length of the left spicule (3.05 mm), but this species was not completely described and more comparisons between them were not possible. Moreover, females of *G*. (*G*.) *primatum* sp. nov. have protruding vulvar lips, while the females of the five above-mentioned species have slightly prominent or non-protruding vulvar lips. The remaining *Gongylonema* species in other hosts differ from the new species in the length of males and left spicule, excepting *G*. *dupuisi* Quentin, 1965 that had similar body length of males and females, and left spicule, but differed in the gubernaculum length (62–87 vs. 160 μm) and number of precloacal papillae (6–8 vs. 4). *Gongylonema graberi* Barré, 1980 had similar morphometrics as *G*. (*G*.) *primatum* sp. nov., although it was reported in a phylogenetically distant host (i.e., *Gallus gallus domesticus* Linnaeus, 1758), without a molecular analysis.

**Figure 6:**
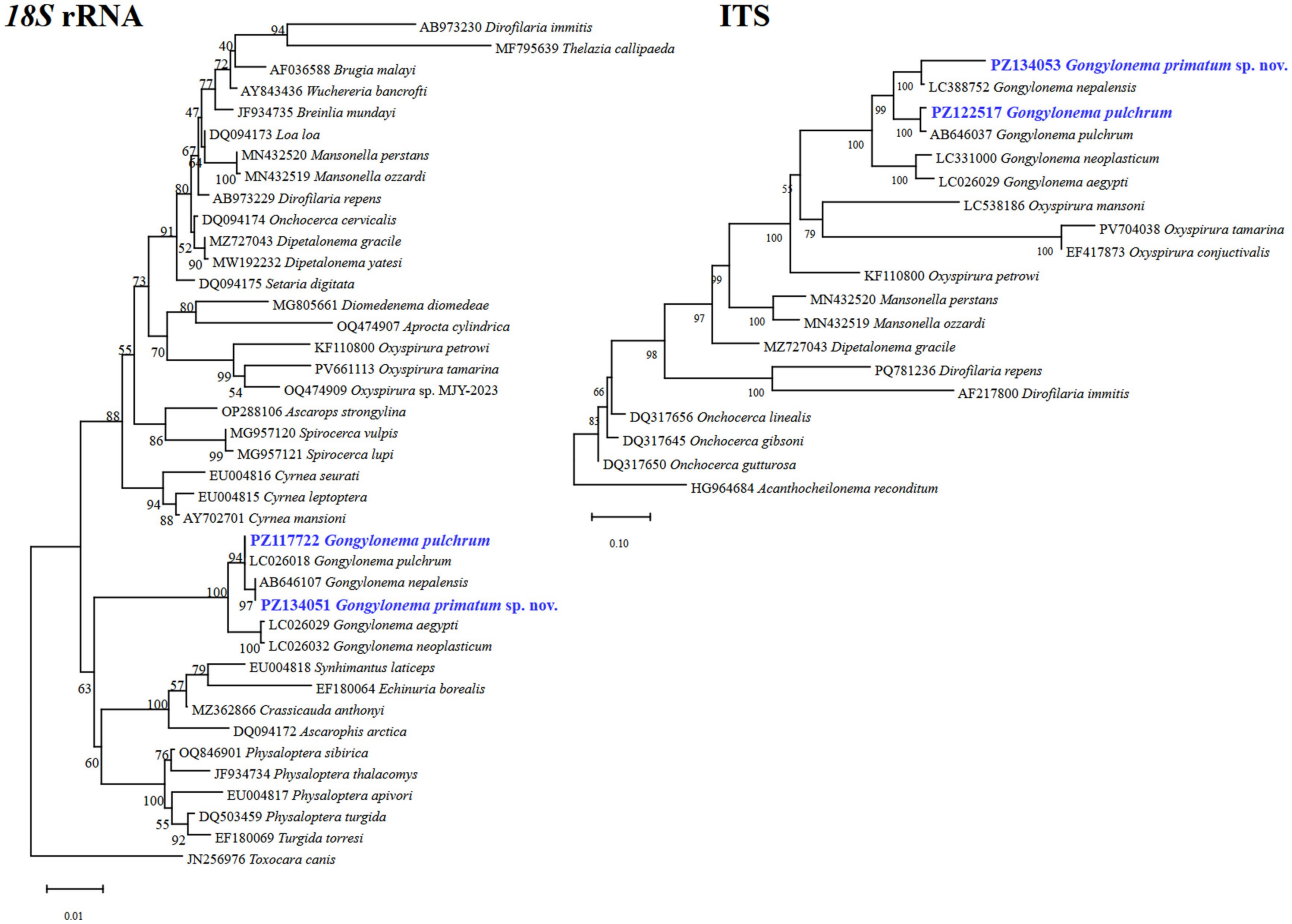
Phylogenetic trees of *Gongylonema* (*G*.) *primatum* sp. nov. and *Gongylonema pulchrum* and their related spirurid species, based on *18S* rRNA and ITS region sequences. Names highlighted in bold blue represent the sequences obtained in the present study. The numbers on phylogenetic trees are bootstrap values based on 1,000 replications, while those before each nematode species are GenBank accession numbers.

**Figure 7:**
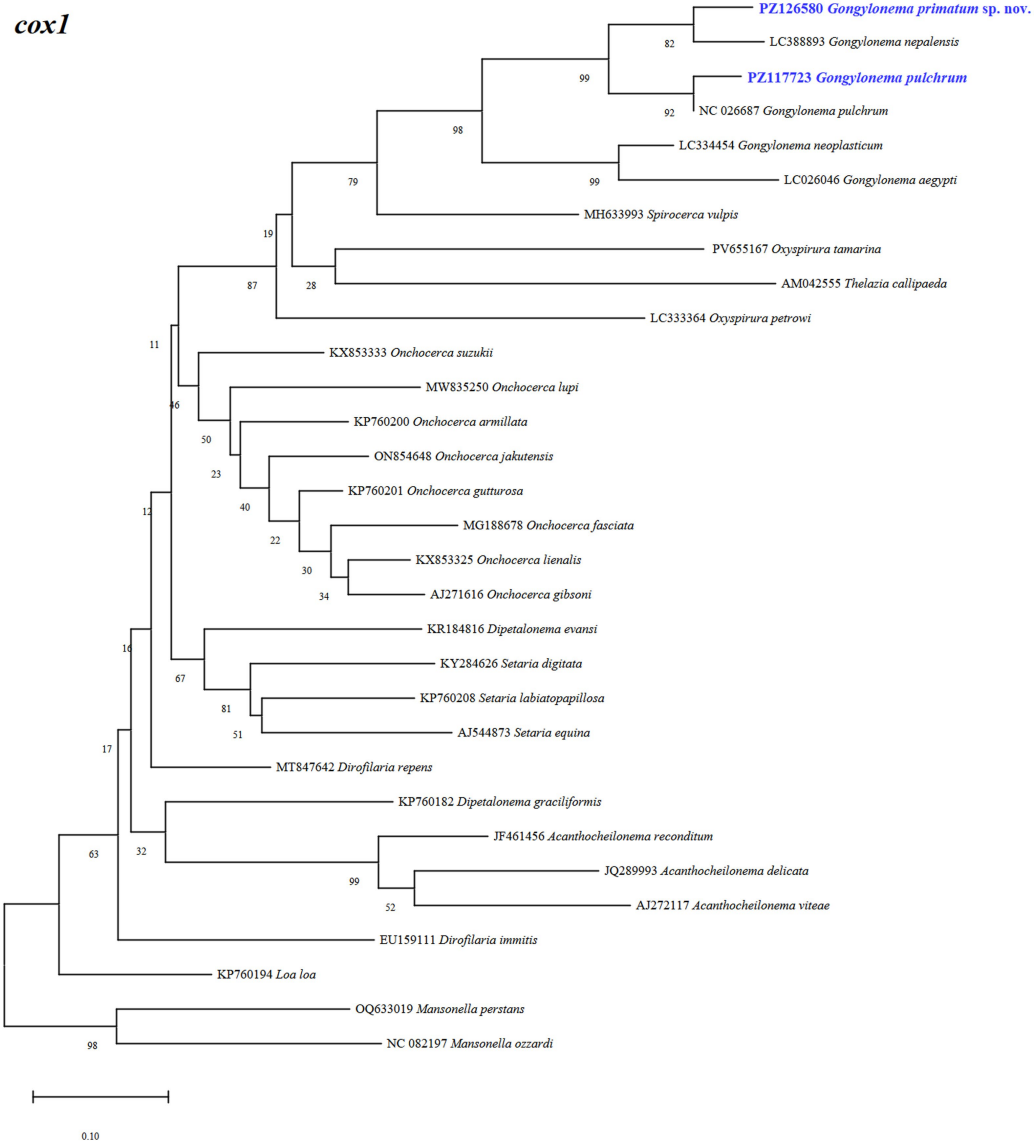
Phylogenetic tree of *Gongylonema* (*G*.) *primatum* sp. nov. and *Gongylonema pulchrum* and their related spirurid species, based on *cox1* sequences. Names highlighted in bold blue represent the sequence obtained in the present study. The numbers on phylogenetic trees are bootstrap values based on 1,000 replications, while those before each nematode species are GenBank accession numbers.

The individual female from *M. sylvanus* was molecularly identified as *G. pulchrum* after comparing our *18S* rRNA (GenBank accession number: PZ117722, 1,068 bp), *28S* rRNA (PZ117695, 687 bp), and ITS (PZ122517, 810 bp) sequences with several sequences from GenBank in various hosts worldwide, which were 100% similar to *G. pulchrum* in the oral cavity of *Homo sapiens* from Slovenia (LR215834), and in the esophagus of cattle *Bos taurus* Linnaeus, 1758 from Mongolia (LC026019). Our *18S* rRNA sequence (PZ117722) clustered with *G. pulchrum* in *Bos taurus* from Mongolia (LC026018) and formed a sister clade with *G. nepalensis* Setsuda, Da, Hasegawa, Behnke, Rana, Dhakal, et Sato, 2016 in the water buffalo *Bubalus bubalis* (Linnaeus, 1758) from Nepal (AB646107), and the present *G*. (*G*.) *primatum* sp. nov. (PZ134051) (Figure 6). The ITS sequence (PZ122517) formed a group with other *Gongylonema* species and more closely with *G. pulchrum* in *Cervus nippon centralis* Kishida, 1936 from Japan (AB646037). For *cox1*, the sequence (PZ117723; 281 bp) showed 99.64–96.80% similarity with *G. pulchrum* in *Bos taurus* from Mongolia (LC026041) and in *Cervus nippon centralis* from Japan (LC388902). In the phylogenetic tree, our sequence grouped with *G. pulchrum* in the goat (probably *Capra hircus*) from China (NC026687) (Figure 7).

The present sequences of all other nematodes from non-human primates (two *S. labiatus*, one *S. midas*) and anteaters (two *T. tetradactyla*), at all markers, were 100% similar to each other. The eight sequences of *P. pithecia* were obtained only by *cox1* and were also similar to all other sequences. For the nearly complete *18S* rRNA gene (PZ134051; 1,693 bp), our sequence was 99.88–100% similar to *G. nepalensis* found in the esophagus of *Bubalus bubalis* from Nepal (AB646107), 99.76–99.82% similar to *G. pulchrum* in the esophagus of *Bos taurus* from Mongolia (LC026018), and formed a group with these two species (Figure 6), as well as being 96.99% similar to *G*. *aegypti* Ashour et Lewis, 1986 in the stomach of the Eastern spiny mouse *Acomys dimidiatus* (Cretzschmar, 1826) from Egypt (LC026029), and 96.93% similar to *G*. *neoplasticum* (Fibiger et Ditlevsen, 1914) in the stomach of the brown rat *Rattus norvegicus* (Berkenhout, 1769) from Japan (LC026032). Our representative partial sequence of the *28S* rRNA gene (PZ134052; 726 bp) was also 100% identical to *G. nepalensis* (AB646107), 99.86% similar to *G. pulchrum* (LC026018), 99.17% similar to *G*. *neoplasticum* (LC026032), and 99.03% similar to *G*. *aegypti* (LC026029).

At the ITS region, our sequence (PZ134053; 1,106 bp) was 97.33–97.43% similar to *G. nepalensis* in the esophagus of *Bos taurus* (LC388752) or oral mucosa of the red fox *Vulpes vulpes* (Linnaeus, 1758) (LC388752), both from Italy, and formed a clade with the latter (Figure 6), 89.12–96.53% similar to *G. pulchrum* in the esophagus of the Honshü sika deer *Cervus nippon centralis* Kishida, 1936 from Japan (AB646037), 96.44% (query cover 69%) similar to *G*. *aegypti* in the stomach of *Acomys dimidiatus* (Cretzschmar, 1826) from Egypt (LC026029), and 96.99% (query cover 69%) similar to *G*. *neoplasticum* in the stomach of *Rattus norvegicus* Berkenhout, 1769 from Japan (LC026032).

The *cox1* gene sequence (PZ126580; 903 bp) was 93.16–93.40% (query cover 94%) similar to *G. nepalensis* in the oral mucosa of *Vulpes vulpes* from Italy (LC388893), 90.40–91.42% (query cover 93–100%) similar to *G. pulchrum* in the esophagus of the Japanese boar *Sus scrofa leucomystax* Temminck, 1842 from Japan (LC388911), 87.38–87.62% (query cover 94%) similar to *G*. *neoplasticum* in *Rattus norvegicus* from Indonesia (LC334451), or 87.23% (93% query cover) similar to *G*. *aegypti* in the stomach of *Acomys dimidiatus* from Egypt (LC026046). In the phylogenetic tree, species of *Gongylonema* are grouped together in a separate clade from the other spirurid species, while the new species formed a cluster with *G. nepalensis* (LC388893) (Figure 7).

## Discussion

The presence of numerous cuticular bosses in the anterior end of the body, dissimilar and unequal spicules, and a gubernaculum [see (23)] allowed us to recognize all the present nematodes as members of the genus *Gongylonema*. *Gongylonema* (*G*.) *primatum* sp. nov. is the sixth species of the genus in non-human primates and the first host record in *P. pithecia*, *S. labiatus*, *S. midas*, and *T. tetradactyla*. To date, there is a sole record of *Gongylonema* sp. in *P. pithecia* from a zoological garden in the Czech Republic [see (24)], and it might be conspecific with the new species, but since information on the genetic analysis of the unnamed species is missing, we refrain from naming it.

This is also the first record of the zoonotic *G. pulchrum* in *M. sylvanus* since it was previously found in *M. fuscata fuscata* (Blyth, 1875), *M*. *f*. *yakui* Kuroda, 1941 (5, 25, 26), and *M. thibetana* Milne-Edwards, 1870 (27). *Gongylonema pulchrum* is a cosmopolitan nematode with apparent low host specificity since it has been reported in various hosts, including non-human primates of the genera *Ateles* Geoffroy, 1806, *Cebus* Erxleben, 1777, and *Macaca* Lacépède, 1799 [see (28)], and seems to occur frequently in non-human primates of the family Callitrichidae [see (10)]. Multi-host infections have already been reported; for example, Lubimov (29) found *G*. *macrogubernaculum* in three non-human primate species (*Cebus hypoleucus* Geoffroy, 1812, *Miopithecus talapoin* Schreber, 1774, and *Macaca mulatta* Zimmermann, 1780) from the Moscow Zoopark. Even though humans are considered accidental hosts of *Gongylonema* species, they are susceptible to being infected by these nematodes [see (30)], as in the case of *G. pulchrum*, which is considered zoonotic (12). We do not know if the new species is zoonotic or not, but it behaves in the same way as *G. pulchrum*, infecting non-human primates, and thus it might be potentially of zoonotic concern. Therefore, special attention should be paid to the occurrence of this nematode species in non-human primates, as well as to monitoring the presence of intermediate hosts around the captive facilities to elucidate its transmission pathways.

Only 4 out of the 15 host species examined were found parasitized in the present study, with a variable number of individual nematodes per infected host. Despite the low number of nematodes in *S. labiatus* and *S. midas*, at least one specimen was used for morphology and one for molecular analysis, thus validating the taxonomic identity of each nematode per host species. We might be overlooking the intraspecific variability of the nematodes in these two host species, but their morphological and molecular similarity with conspecific individuals collected in *P. pithecia* and *T. tetradactyla* from other hosts was practically the same, and they were considered as belonging to *G*. (*G*.) *primatum* sp. nov.

The present nematodes showed intraspecific variability in the number of precloacal papillae, mostly with unequal numbers of papillae on both sides of the caudal end of males. This variability was even observed among the nematodes from the same host, as in the case of those in *T. tetradactyla* and *P. pithecia* that showed the following combinations of papillae (right/left): 6/6, 6/7, 6/8, 7/6, 7/7 and 5/5, 5/6, 6/6, 7/6, respectively (Figures 4B, 5A–G). This feature should be further corroborated in other known and described species, since there is a clear differentiation between the number of papillae on the right and left sides, whereas postcloacals are more consistent in number, although those near the tip of the tail are very small and hard to see. The variability in the number of caudal papillae seems to be a common phenomenon in other *Gongylonema* species, such as in *G*. *neoplasticum,* as reported by Kruidenier and Peebles (31), and Eira et al. (32), who reported 6–9 and 8–9 pairs of caudal papillae, respectively. Another variable measurement was the body length, which showed differences in the specimens depending on the host species, with those in *P. pithecia* being larger than in other hosts (see Table 2). The development of parasitic nematodes depends on the host type, and in the present case, *P. pithecia* appears to be a more suitable host for *G*. (*G*.) *primatum* sp. nov. The role of this non-human primate should be studied in captivity, since it might represent the main source of infection by *Gongylonema* in other hosts.

On the other hand, the length of the left spicule seems to be a reliable feature to distinguish species of *Gongylonema*, as already stated by Lichtenfels (33) because the body and right spicule lengths are very variable, both intra- and interspecifically. A good example of this is the tentative allocation of the nematodes found in the oesophageal mucosa of the water buffalo *Bubalus bubalis* (Linnaeus, 1758) from Nepal as *G. pulchrum*, after comparing them with nematodes in other ruminants and identifying similarities in almost all measurements (except left spicule length) [see (34)]. These nematodes were later sequenced and found to represent a new species (*G. nepalensis*) [see (35)], thus showing that the original differences in the left spicule length would be enough to consider it as a distinct species.

The apparent close relationships of the newly obtained sequences of *G*. (*G*.) *primatum* sp. nov. at *18S* rRNA and *28S* rRNA genes with *G*. *aegypti*, *G*. *neoplasticum*, *G. nepalensis*, and *G. pulchrum* clearly showed that both markers have low resolution for distinguishing between these nematodes, as already stated by Chan et al. (36) and Mejías-Alpízar et al. (37). However, the four above-mentioned *Gongylonema* species showed higher divergence (> 2%) at ITS and *cox1* loci, thus elucidating the suitability of both markers in distinguishing a new species from its congeners. Unfortunately, none of the species reported in non-human primates (i.e., *G*. *capucini*, *G*. *macrogubernaculum*, *G*. *microgubernaculum*, *G. pulchrum*, and *G*. *saimirisi*) have so far been molecularly analyzed, so their comparison with the new species is not possible.

A suggested practical diagnosis for the presence of *Gongylonema* should first include the detection of eggs or first-stage larvae in smears from the buccal cavity, tongue, and/or anterior region of the digestive tract by light microscopy and then be confirmed by molecular analysis. In the case that the latter method is not available, one useful morphological feature to distinguish between larvae is the presence of spine-like mucrons on the tail tip of unsheathed and manually released larvae and of cuticular bosses in the anterior body region. It is important to note that mucrons on the tail tip are mainly present in first-stage larvae, although early second-stage larvae might also bear these structures. The presence of mucrons on the tail tip seems to be very common, and it has been shown in larvae from congeners, like in *G*. *dupuisi* [see p. 65 in (38)], and *G. pulchrum* [see Figures 171 (5), 173 (4–6) in (39)]. Occasionally, when coinfections between *Gongylonema* and *Oxyspirura* occur, the distinction between eggs and larvae is complicated since they are very similar, but first-stage larvae of *Oxyspirura* lack the mucrons on the tail tip [see (40)]. Special attention should be paid during the flotation/centrifugation methods of fecal samples and the use of light microscopy since the occurrence of developmental stages might be overlooked in low-load infections and because the method is not sensitive enough (per. obs.). If an infection with *Gongylonema* is suspected, it is necessary to monitor and examine the buccal cavity and tongue through smears.

The role of *Gongylonema* nematodes as responsible for the mortality of some hosts in the present study was not elucidated. Gongylonemosis is usually non-pathogenic in natural hosts, although a lingual form was associated with increased salivation, facial swelling, and systemic pasteurellosis in Goeldi’s marmoset *Callimico goeldii* Thomas, 1904 (6). Apparently, the infection caused by these nematodes might cause discomfort and stress in non-human primates, thus leading to self-inflicted wounds and the entrance of secondary infections (7).

The infection source is unknown, but hosts might have been parasitized prior to their importation to the Czech Republic or after introduction and interaction with other hosts in their current enclosures. As in other *Gongylonema* species, cockroaches or dung beetles might act as intermediate hosts (41) and may represent the main route of infection since they freely occur around the facilities and are easy prey for the non-human primates. Unfortunately, we were not aware of the husbandry conditions, enclosure hygiene, or insect control within the facilities, but we believe that control of pests (e.g., cockroaches), prophylactic anthelmintic practices, and hygiene (avoiding fecal contamination of the ground) are mandatory in all facilities to avoid the spread of these infections and possible loss of animals.

The use of morphology and, especially, the molecular analysis helped in clarifying the identity of the nematodes despite their intraspecific variability. As Setsuda et al. (35) stated, the accumulation of genetic data on diverse *Gongylonema* species could be useful for understanding the relationships of the species within the genus and, in this case, for elucidating the identity of the specimens.

Recently, an *Oxyspirura* species was reported in three tamarin monkey species, including *S. midas*, from a zoological garden in the Czech Republic (40). The same non-human primate specimen is herein reported as a host of *G*. (*G*.) *primatum* sp. nov., thus showing that apparently the occurrence of nematodes of these two genera in captive animals is common and should be frequently monitored to avoid acute infections or potential death during trade or translocations of animals. Several members of *Gongylonema* and *Oxyspirura* are rightfully classified as zoonotic (e.g., *G. pulchrum*, *O*. *mansoni*) after being transferred from animals to humans, even though the transmission patterns are unknown [see (42)]. Therefore, further research and continuous monitoring on the occurrence of these parasites in caged or cultured animals is mandatory since they are commonly handled by humans, might facilitate transmission, and to control the occurrence of potential intermediate hosts. Moreover, several invertebrate hosts, which normally live around humans (e.g., cockroaches, grasshoppers), are used as intermediate hosts and might represent a route of transmission.

## Data Availability

The sequences generated in the present study were submitted to the GenBank database under the accession numbers: *18S* rRNA (PZ117722, PZ134051), *28S* rRNA (PZ117695, PZ134052), *cox1* (PZ117723, PZ126580) and ITS (PZ122517, PZ134053).

## Footnotes

1
https://www.ncbi.nlm.nih.gov

2
https://blast.ncbi.nlm.nih.gov/Blast.cgi

## References

[1] da Costa-Cordeiro H de Vasconcelos Melo FT Giese EG Santos JND. *Gongylonema* parasites of rodents: a key to species and new data on *Gongylonema neoplasticum*. J Parasitol. (2018) 104:51–9. doi: 10.1645/17-3, 29135391

[2] Kinsella JM Robles MDR Preisser WC. A review of *Gongylonema* spp. (Nematoda: Gongylonematidae) in North American rodents with description of a new species from the cotton rat, *Sigmodon hispidus* (Mammalia: Cricetidae). Zootaxa. (2016) 4107:277–84. doi: 10.11646/zootaxa.4107.2.9, 27394819

[3] Kramar U Skvarč M Logar M Islamović S Kolenc M Šoba B. First case of human *Gongylonema pulchrum* infection in Slovenia. J Helminthol. (2019) 94:e62. doi: 10.1017/S0022149X19000658, 31328705

[4] Adkesson MJ Langan JN Paul A. Evaluation of control and treatment of *Gongylonema* spp. infections in callitrichids. J Zoo Wildl Med. (2007) 38:27–31. doi: 10.1638/06-005.1, 17469272

[5] Brack M. Gongylonematiasis in the common marmoset (*Callithrix jacchus*). Lab Anim Sci. (1996) 46:266–70. 8799930

[6] Craig LE Kinsella JM Lodwick LJ Cranfield MR Strandberg JD. *Gongylonema macrogubernaculum* in captive African squirrels (*Funisciurus substriatus* and *Xerus erythropus*) and lion-tailed macaques (*Macaca silenus*). J Zoo Wildl Med. (1998) 29:331–7. doi: 10.2307/20095776, 9809609

[7] Duncan M Tell L Gardiner CH Montali RJ. Lingual gongylonemiasis and pasteurellosis in Goeldi's monkeys (*Callimico goeldi*). J Zoo Wildl Med. (1995) 26:102–8. doi: 10.2307/20095443

[8] Oliveira AR Souza TD Flecher MC Gardiner CH Santos RL. First report of *Gongylonema* sp. in a free ranging callitrichid from the Brazilian Atlantic Forest: case report. Arq Bras Med Vet Zootec. (2019) 71:777–81. doi: 10.1590/1678-4162-10760

[9] Sato H Une Y Takada M. High incidence of the gullet worm, *Gongylonema pulchrum*, in a squirrel monkey colony in a zoological garden in Japan. Vet Parasitol. (2005) 127:131–7. doi: 10.1016/j.vetpar.2004.10.005, 15631906

[10] Uni S Abe M Harada K Kaneda K Kimata I Abdelmaksoud NM . New record of *Gongylonema pulchrum* Molin, 1857 from a new host, *Macaca fuscata*, in Japan. Ann Parasitol Hum Comp. (1992) 67:221–3. doi: 10.1051/parasite/1992676221

[11] Wong MM Conrad HD. Prevalence of metazoan parasite infections in five species of Asian macaques. Lab Anim Sci. (1978) 28:412–6. 100650

[12] Kurumanastirli B Yılmaz YA. Literature review of *Gongylonema pulchrum*: a rare nematode. Turk Parazitol Derg. (2021) 45:311–6. doi: 10.4274/tpd.galenos.2021.77486, 34889200

[13] Hodda M. Phylum Nematoda: a classification, catalogue and index of valid genera, with a census of valid species. Zootaxa. (2022) 5114:1–289. doi: 10.11646/zootaxa.5114.1.1, 35391386

[14] Baylis HA. On the species of *Gongylonema* (Nematoda). J Comp Pathol Ther. (1925) 38:46.

[15] Chabaud AG. "Spirurida: Spiruroidea, Habronematoidea and Acuarioidea". In: Anderson RC Chabaud AG Willmott S, editors. Keys to the Nematode Parasites of Vertebrates: Archival Volume. Wallingford: CAB International (2009). p. 361–90.

[16] Halajian A Eslami A Salehi N Ashrafi-Helan J Sato H. Incidence and genetic characterization of *Gongylonema pulchrum* in cattle slaughtered in Mazandaran Province, northern Iran. Iran J Parasitol. (2010) 5:10–8. 22347239 PMC3279837

[17] Kalyanasundaram A Blanchard KR Henry C Brym MZ Kendall RJ. Phylogenetic analysis of eyeworm (*Oxyspirura petrowi*) in northern bobwhite (*Colinus virginianus*) based on the nuclear 18S rDNA and mitochondrial cytochrome oxidase 1 gene (COX1). Parasitol Open. (2018) 4:1–7. doi: 10.1017/pao.2018.2

[18] Allen JD Esquela-Kerscher A. Short report: *Gongylonema pulchrum* infection in a resident of Williamsburg, Virginia, verified by genetic analysis. Am J Trop Med Hyg. (2013) 89:755–7. doi: 10.4269/ajtmh.13-0355, 23958907 PMC3795108

[19] Bowles J Blair D McManus DP. Genetic variants within the genus *Echinococcus* identified by mitochondrial DNA sequencing. Mol Biochem Parasitol. (1992) 54:165–73. doi: 10.1016/0166-6851(92)90109-W, 1435857

[20] Casiraghi M Anderson TJ Bandi C Bazzocchi C Genchi C. A phylogenetic analysis of filarial nematodes: comparison with the phylogeny of *Wolbachia* endosymbionts. Parasitology. (2001) 122:93–103. doi: 10.1017/S0031182000007149, 11197770

[21] Kumar S Stecher G Suleski M Sanderford M Sharma S Tamura K. MEGA12: molecular evolutionary genetic analysis version 12 for adaptive and green computing. Mol Biol Evol. (2024) 41:263. doi: 10.1093/molbev/msae263, 39708372 PMC11683415

[22] ICZN (International Commission on Zoological Nomenclature). Amendment of articles 8, 9, 10, 21 and 78 of the international code of zoological nomenclature to expand and refine methods of publication. Bull Zool Nomencl. (2012) 219:1–10. doi: 10.3897/zookeys.219.3994, 22977348 PMC3433695

[23] Chabaud AG. "Spirurida". In: Anderson RC Chabaud AG Willmot S, editors. Keys to the Nematode Parasites of Vertebrates. Farnham Royal, Bucks, England: CAB International (2009).

[24] Zoo Jihlava. The Annual Report 2020. Nomascus Zoo Jihlava. Jihlava, Czech Republic: Zoo Jihlava (2020) (In Czech).

[25] Setsuda A Varcasia A Scala A Ozawa S Yokoyama M Torii H . *Gongylonema* infection of wild mammals in Japan and Sardinia (Italy). J Helminthol. (2018) 94:e13. doi: 10.1017/S0022149X18001001, 30457072

[26] Uni S Kobayashi S Miyashita M Kimura N Kato A Aimi M . Geographic distribution of *Gongylonema pulchrum* and *Gongylonema macrogubernaculum* from *Macaca fuscata* in Japan. Parasite. (1994) 1:127–30. doi: 10.1051/parasite/1994012127, 9140479

[27] Zhu Y Ji H Li J-H Xia D-P Sun B-H Xu Y-R . First report of the wild Tibetan macaque (*Macaca thibetana*) as a new primate host of *Gongylonema pulchrum* with high incidence in China. J Anim Vet Adv. (2012) 11:4514–8.

[28] Yamashita J. Ecological relationships between parasites and primates: I. Helminth parasites and primates. Primates. (1963) 4:1–96. doi: 10.1007/BF01659699

[29] Lubimov MP. *Gongylonema macrogubernaculum* n. sp. (nematode) from monkeys. Parasitology. (1931) 23:446–8. doi: 10.1017/S0031182000013846

[30] Waisberg V dos Santos LW Vasconcelos-Santos DV. Intraocular *Gongylonema* infection: first case in humans. Ocul Immunol Inflamm. (2018) 26:595–7. doi: 10.1080/09273948.2016.1232739, 27726517

[31] Kruidenier FJ Peebles CR. *Gongylonema* of rodents: *G. neoplasticum* (redefinition); *G. dipodomysis* n. sp.; and *G. peromysci* n. sp. Trans Am Microsc Soc. (1958) 77:307–15. doi: 10.2307/3223695

[32] Eira C Miquel J Vingada J Torres J. Natural infection of *Oryctolagus cuniculus* (Lagomorpha, Leporidae) by *Gongylonema neoplasticum* (Nematoda, Gongylonematidae) in Portugal. Acta Parasitol. (2006) 51:119–22. doi: 10.2478/s11686-006-0018-4

[33] Lichtenfels JR. Morphological variation in the gullet nematode, *Gongylonema pulchrum* Molin, 1857, from eight species of definitive hosts with a consideration of *Gongylonema* from *Macaca* spp. J Parasitol. (1971) 57:348–55. doi: 10.2307/3278041, 5553452

[34] Makouloutou P Rana HB Adhikari B Devkota B Dhakal IP Sato H. A distinct genetic population of *Gongylonema pulchrum* from water buffaloes in Nepal. J Parasitol. (2013) 99:669–76. doi: 10.1645/12-143.1, 23421498

[35] Setsuda A Da N Hasegawa H Behnke JM Rana HB Dhakal IP . Intraspecific and interspecific genetic variation of *Gongylonema pulchrum* and two rodent *Gongylonema* spp. (*G. aegypti* and *G. neoplasticum*), with the proposal of *G. nepalensis* n. sp. for the isolate in water buffaloes from Nepal. Parasitol Res. (2016) 115:787–95. doi: 10.1007/s00436-015-4806-3, 26531300

[36] Chan AHE Chaisiri K Saralamba S Morand S Thaenkham U. Assessing the suitability of mitochondrial and nuclear DNA genetic markers for molecular systematics and species identification of helminths. Parasites Vectors. (2021) 14:233. doi: 10.1186/s13071-021-04737-y, 33933158 PMC8088577

[37] Mejías-Alpízar MJ Porras-Silesky C Rodríguez EJ Quesada J Alfaro-Segura MP Robleto-Quesada J . Mitochondrial and ribosomal markers in the identification of nematodes of clinical and veterinary importance. Parasites Vectors. (2024) 17:77. doi: 10.1186/s13071-023-06113-4, 38378676 PMC10880205

[38] Quentin J-C. Cycle biologique de *Gongylonema dupuisi* Quentin, 1965 Nematoda Spiruridae. Ann Parasitol Hum Comp. (1969) 44:59–67. doi: 10.1051/parasite/1969441059, 5392664

[39] Skrjabin KI Sobolev AA Ivashkin VM. Principles of Nematodology, Volume XVI. Spirurata of Animals and man and the Diseases Caused by them. Part 4: Thelazioidea. Moscow: Akademia Nauk SSSR (1967) (in Russian).

[40] Máca O González-Solís D. A new *Oxyspirura* (Nematoda, Thelaziidae) in three captive non-human primate species. Front Vet Sci. (2025) 12:1650452. doi: 10.3389/fvets.2025.1650452, 40979364 PMC12445999

[41] Cappucci DT Jr Augsburg K Klinck PC. "Gongylonemiasis". In: Schultz MG, editor. CRC Handbook Series in Zoonoses. Section C: Parasitic Zoonoses (1982).

[42] Doanh P Hien H Dung B Nawa Y. First confirmation of the chicken eyeworm, *Oxyspirura mansoni*, as a causative pathogen of human cutaneous larva migrans by morphological and molecular evidence. Parasitol Res. (2025) 124:46. doi: 10.1007/s00436-025-08473-5, 40304770 PMC12043789

